# GnRH-agonist pretreatment in hormone replacement therapy improves pregnancy outcomes in women with male-factor infertility

**DOI:** 10.3389/fendo.2022.1014558

**Published:** 2022-09-23

**Authors:** Juanjuan Yu, Peiqin Chen, Yifan Luo, Mu Lv, Liqun Lou, Qimeng Xiao, Luxia Wang, Juan Chen, Mingzhu Bai, Zhenbo Zhang

**Affiliations:** ^1^Department of Obstetrics and Gynecology, Reproductive Medicine Centre, Shanghai General Hospital, Shanghai Jiao Tong University, Shanghai, China; ^2^Department of Obstetrics and Gynecology, the International Peace Maternity & Child Health Hospital of China Welfare Institute, Shanghai Jiao Tong University, Shanghai, China; ^3^Department of Obstetrics and Gynecology, Zhongshan Wusong Hospital, Fudan University, Shanghai, China; ^4^Centre for Reproductive Medicine, Xuzhou Maternity and Child Health Care Hospital, Jiangsu, China; ^5^Shanghai Key Laboratory for Assisted Reproduction and Reproductive Genetics, Renji Hospital, Shanghai Jiao Tong University, Shanghai, China

**Keywords:** male-factor infertility, frozen embryo transfer, endometrial preparation, gonadotropin-releasing hormone agonist, hormone replacement treatment, inverse probability of treatment weighting, live birth rate

## Abstract

**Objective:**

This study aimed to examine the efficacy of HRT with gonadotropin-releasing hormone agonist (GnRH-a) pre-treatment in women with male-factor infertility who underwent a frozen embryo transfer (FET) programme.

**Design:**

Between January 2016 and October 2020, 2733 women with male-factor infertility who underwent the HRT protocol as the endometrial preparation method were enrolled at two Reproductive Medicine Centres. Patients were divided into two groups based on whether they had GnRH-a pre-treatment before HRTs: the GnRHa-HRT group and the HRT group. The inverse probability of treatment weighting (IPTW) method was conducted to balance patient baseline characteristics between treatment cohorts to reduce selection bias. The live birth rate was considered regarded as the primary pregnancy outcome.

**Results:**

Multivariate logistic regression adjusted for confounding factors, the GnRHa-HRT group showed a notably higher rate of live birth (OR 2.154, 95% CI 1.636~2.835, P<0.001) when compared to the HRT group. Additionally, the rate of miscarriage was significantly lower in the GnRHa-HRT group. The GnRHa-HRT group had significantly higher rates of biochemical pregnancy, clinical pregnancy, multiple pregnancy, and term birth.

**Conclusion:**

The endometrial preparation protocol of HRT with GnRH-a pre-treatment could obviously increase the live birth rate for women with male-factor infertility undergoing the FET programme.

## Introduction

Infertility is one of the major health problems worldwide, affecting 8-12% of couples at reproductive ages (1–3). According to the World Health Organization, 50% of couples have low fertility due to male-factors (4, 5). Successful pregnancy in couples with male infertility often requires embryo transfer, including fresh embryo transfer and frozen embryo transfer (FET). Recently, improvements in cryopreservation techniques (vitrification) and the development of effective ovarian stimulation protocols have markedly increased options for elective FET protocols (6, 7). FET cycle practice facilitates the elective single-embryo transfer and lessens the effect of steroid hormones used for ovarian stimulation and embryo at the time of egg collection when compared to fresh embryo transfer (8). At the same time, it gives endometrial receptivity enough time to be regulated. And a series of studies have indicated that pregnancy rates with FET procedures in assisted reproductive patients are higher than those with fresh cycle procedures (9–11). Therefore, the use of FET is being used more widely.

During a FET cycle, the determination of the appropriate endometrial preparation protocol is critical to maximizing the success of assisted reproduction technology (ART). There are three common methods of endometrial preparation: hormone replacement therapy (HRT), natural cycle, and stimulation cycle. HRTs are currently the most widely utilized method due to its wide range of applications, no frequent follow-up, and low cycle cancellation rate. Patients with or without normal ovaries and with or without normal menstrual cycles can participate in the HRT program (12). Several studies have concluded that HRT cycles are comparable to natural cycles in terms of pregnancy outcomes (13, 14).

To avoid hormone disruptors in subsequent therapy when using HRTs, gonadotropin-releasing hormone agonist (GnRH-a) pre-treatment can downregulate any hormones produced by the ovaries. However, it is yet unclear whether HRT with GnRH-a pre-treatment can improve reproductive outcomes. A recent clinical study suggests that pre-treatment of GnRH-a significantly improves the live birth rate in patients with multiple failed embryo implantation (15). Moreover, some studies indicate that the use of GnRH-a can improve endometrial receptivity (16–18). Studies have shown that HRT with GnRH-a pre-treatment improves pregnancy outcomes in patients with endometriosis and adenomyosis (19–21), but not in those with polycystic ovary syndrome (PCOS) (22, 23).

The cases number of endometriosis, adenomyosis, and PCOS infertility are only a part of the population, but male-factors make up the majority of ART patients. However, the specific disease for which the GnRH-a preconditioning HRT regimen is applicable is unclear. And endometrial preparation for male factor infertility has received limited attention. To address this issue, we divided eligible patients with male-factor infertility into the HRT group and the HRT with GnRH-a pre-treated group. Then we analysed the differences in the pregnancy outcomes between the two groups.

## Methods

### Study design and participants

The retrospective cohort study was conducted at two reproductive medicine centres, Shanghai General Hospital and Xuzhou Maternity and Child Health Care Hospital. 2733 cases of endometrial preparation protocols have been analysed for HRTs during the FET programme between 1 January 2016, and 1 October 2020. The two reproduction centres specialize in treating patients with male factor infertility, so there have been many cases with male factor infertility. In this study, we defined male factors infertility as moderate to severe oligo-astheno-terato-spermia and azoospermia. Comprehensive assessment of male factor infertility based on semen analysis. Semen quality data were analysed according to the fifth edition of WHO guideline (24).

The inclusion criteria were as follows:1) A FET programme and endometrial preparation method using HRT protocol with or without GnRH-a. 2) Male-factor is identified as the cause of infertility. 3) The embryos transferred from female patients are of high-quality. The embryos with 7-12 cells and grade I-II were selected at cleavage stage, and the embryos with 4BB, 4BA, 4AB or 4AA grade were selected at blastocyst stage (25–27). 4) Endometrial thickness ≥8mm on the transfer day.

**Figure 1:**
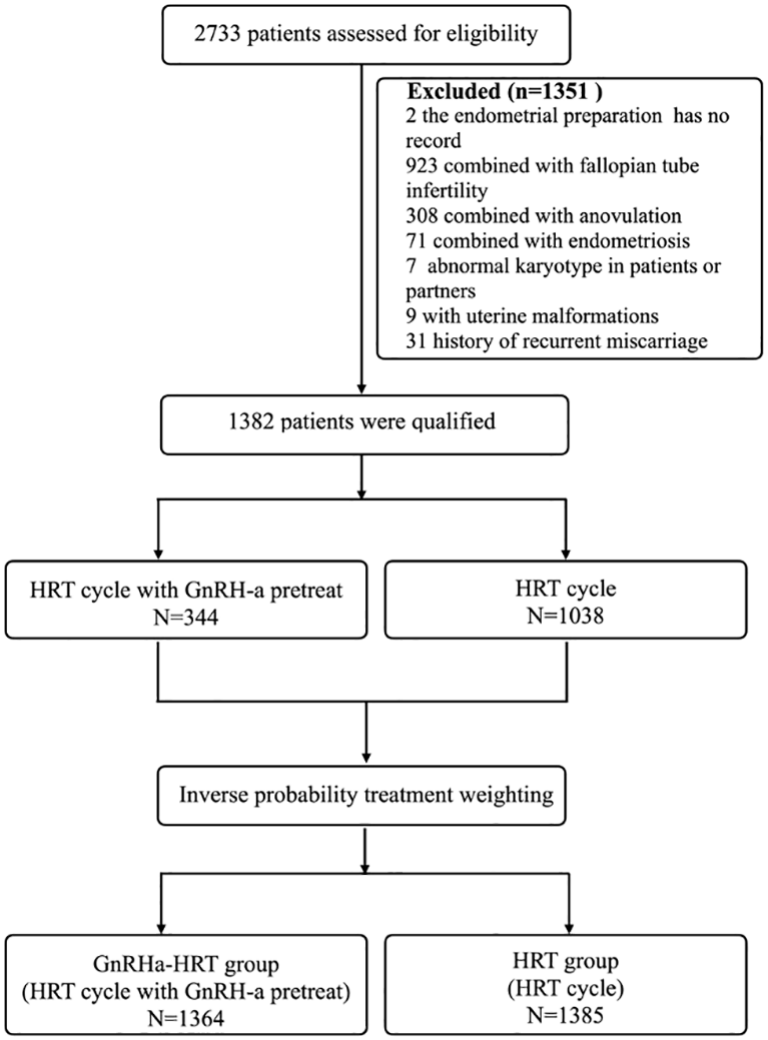
Trial profile. HRT, hormone replacement therapy; GnRH-a, gonadotropin-releasing hormone agonist.

Exclusion criteria were as follows: 1) The woman’s age was<20 or ≥40 years old. 2) The causes of infertility combined with other diseases such as ovulation factors, fallopian tube factors, endometriosis, adenomyosis, etc. 3) Uterine abnormalities in women, such as congenital uterine malformations, fibroids that affect pregnancy, endometrial polyps, etc. 4) Infertile women with recurrent pregnancy loss or recurrent implantation failure history. 5) Any chromosomal abnormality in either spouse. 6) The female patients had a severe chronic or acute systemic disease. A detailed flow chart of patient selection can be found in **Figure 1**.

### Endometrial preparation protocols

According to the indications of endometrial preparation methods and patients’ informed consent, eligible patients were divided into an HRT group (HRT group) and an HRT cycle with GnRH-a pre-treatment group (GnRHa-HRT group).

**Figure 2:**
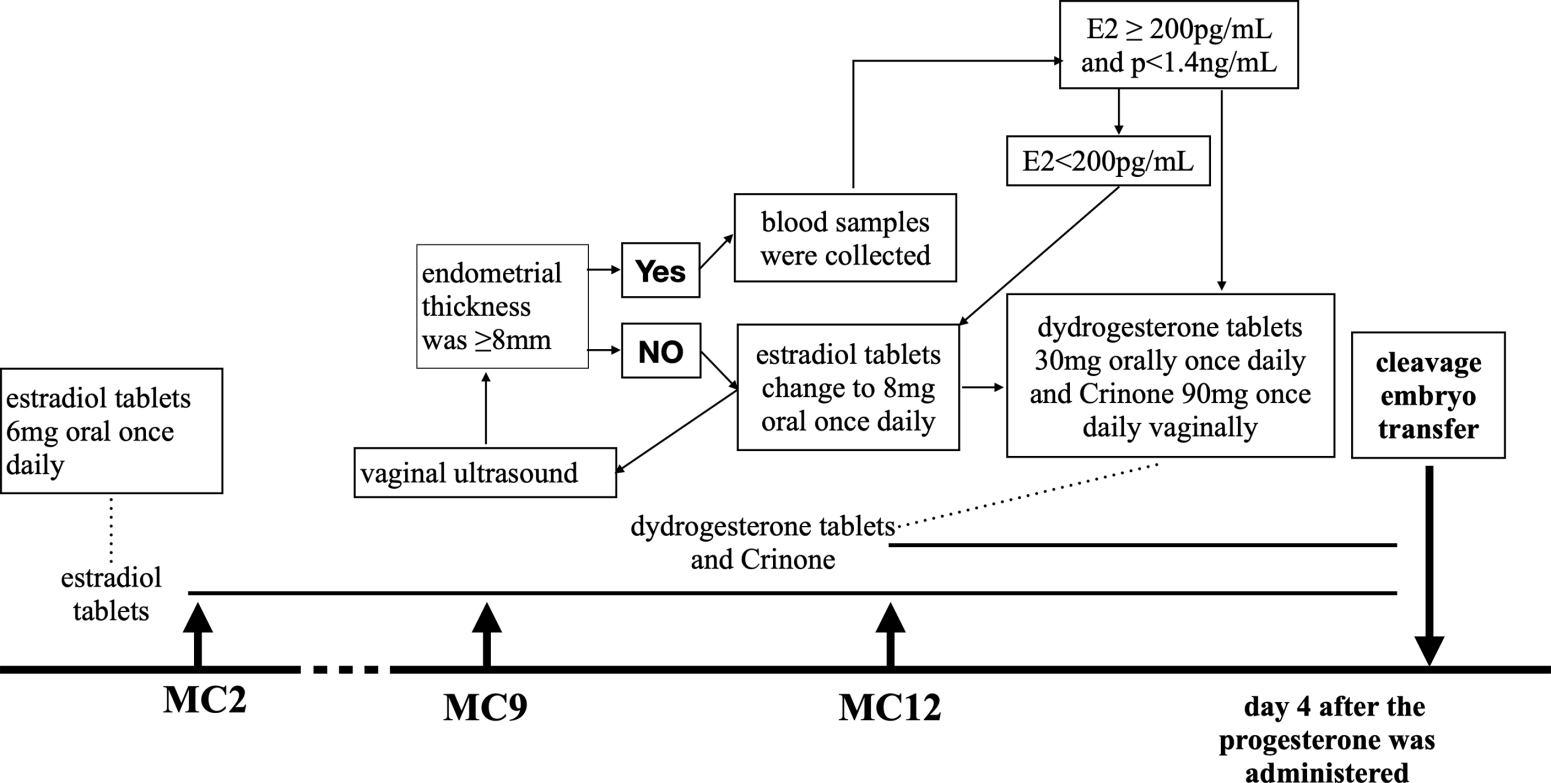
HRT protocol. MC, menstrual cycles.

In the HRT protocols, as shown in **Figure 2**, estradiol tablets (Femoston; Abbott Biologicals B.V., The Netherlands) 6mg once daily oral use from the next day after menstruation. On the 7th day of medication, endometrial thickness was examined by vaginal ultrasound, estradiol tablets were changed to 8 mg/d if necessary, and the duration of medication was extended. Blood samples were collected when the endometrial thickness was ≥8mm. The endometrial transformation was performed when the E2 level was≥ 200pg/mL and the progesterone level was <1.4ng/mL. On day 10 of estrogen medication, dydrogesterone tablets (Duphphaston; Abbott Biologicals B.V., The Netherlands) 30mg orally once daily and progesterone (Crinone; Fleet Laboratories Limited, UK) 90mg once daily vaginally were added. According to the embryo transfer stage, if the cleavage embryo is on day 3, it will be transplanted on day 4 after the progesterone was administered.

In the GnRHa-HRT group, the patient was injected with 3.75mg GnRH-a (Diphereline, Ipsen Pty Ltd., France) on the second day of menstruation, and the HRT protocol will be started on day 28 later.

### Embryo transfer procedure

After endometrium preparation, vitrified embryos were thawed for transfer. In all FET cycles, no more than two embryos were transferred. According to the cryopreserved embryos of the patients, blastocysts transfer was preferred, and cleavage embryo was selected if there was no blastocysts. Assisted hatching is routinely performed before embryo transfer. The embryo transfer was performed *via* the flexible catheter (Frydman,1321600) under transabdominal ultrasound guidance. Luteal support was continued until a negative pregnancy test was obtained on the 14th day after embryo transfer. If pregnancy was achieved, hormone administration continued until 12 weeks’ gestation.

### Definition of pregnancy outcome

Levels of serum human chorionic gonadotropin (β-HCG) serum were determined in all patients on day 14 after embryo transfer. Biochemical pregnancy was taken into account if the serum β-HCG level was ≥5 IU/L. Clinical pregnancy was considered if a gestational sac was found on ultrasound on the 28th day after embryo transfer. Biochemical abortion was defined as positive blood β-hCG 14 days after embryo transfer, but no gestational sac was detected on ultrasound 28 days after embryo transfer. A clinical pregnancy that ends in miscarriage must do so before the 28th week of intrauterine pregnancy, whether spontaneous or therapeutic and foetus’ weight of <1000g. The ratio of live birth cycles to all cycles was defined as the live birth rate. The delivery of any surviving neonate at 28 weeks of gestation and beyond was referred to as a live birth.

### Statistical analysis

All analyses were conducted using R (R Project for Statistical Computing, Austria), v 4.1.2. The inverse probability of treatment weighting (IPTW) approach was utilized for generating a propensity model (28). Individuals are weighted as the inverse of their probability in each group as the predicted probability per sample. The balance of baseline characteristics between groups was evaluated using the standardized mean difference (SMD), with SMD <0.1 considered balanced (29, 30). Univariate and multivariate logistic regression models were used to analyze the relationship between treatment groups and pregnancy outcomes. Multiple logistic regression was performed to balance confounders: maternal age, body mass index, type of infertility, duration of infertility, endometrial thickness, number of embryos transferred, and embryo type. Patient characteristics and pregnancy outcomes were represented as mean ± standard deviation (SD) or number (%) of patients. All P-values are two-tailed. The P-value for statistically significant comparisons was less than 0.05. The IPTW analyses were performed *via* the “RISCA” R package, v1.0.1 (31). P-value and SMD of baseline characteristics were calculated using the “TableOne” R package, v 0.13.2 (32). The function svyglm of the “survey” R package, v 4.1-1, is used to perform univariate and multivariate logistic regression analysis (33). R scripts can be provided if needed.

## Results

2733 patients enrolled in this retrospective study. Of these, 2 cases were lost to follow-up, 923 cases were combined with fallopian tube infertility, 308 cases were combined with anovulation, 71 cases were combined with endometriosis, 7 cases were abnormal karyotypes in patients or partners, 9 cases were with uterine malformations, and 31 cases were history of recurrent miscarriage. 1351 patients were excluded, leaving1382 to be enrolled. Of the total of 1382 women, 1038 patients underwent the HRT group, and 344 underwent the GnRHa-HRT group **(** **Figure 1****).**

### Baseline characteristics of patients

The basic conditions of female patients with male-factor infertility in the HRT group and the GnRHa-HRT group are shown in **Table 1**. Before IPTW, the baseline characteristics of the two groups were statistically different. Female patients in the GnRHa-HRT group were older, had a higher body mass index (BMI), had longer infertility years, and had a thicker endometrium. And there were statistically distinct types and numbers of embryos between the two groups.

**Table 1:** Basic characteristics of unweighted and IPTW study populations.

Characteristics	Unmatched				IPTW			
	HRT	GnRHa-HRT	P	SMD	HRT	GnRHa-HRT	P	SMD
N	1038	344			1385	1364		
Maternal age, mean (SD)	29.61 (3.85)	30.11 (4.07)	0.041	0.126	29.70 (3.90)	29.59 (4.09)	0.691	0.027
Body mass index, mean (SD)	21.68 (2.98)	22.99 (3.16)	< 0.001	0.424	22.06 (3.24)	22.16 (2.91)	0.622	0.032
Endometrial thickness, mean (SD)	9.20 (1.17)	9.38 (1.10)	0.014	0.156	9.24 (1.19)	9.24 (1.10)	0.972	0.002
Duration of infertility, mean (SD)	3.78 (2.87)	4.31 (2.91)	0.003	0.184	3.93 (3.01)	4.04 (2.72)	0.561	0.037
Type of infertility, n (%)			0.189	0.085			0.869	0.011
Primary infertility	790 (76.1)	249 (72.4)			1045.8 (75.5)	1023.7 (75.0)		
Secondary infertility	248 (23.9)	95 (27.6)			339.0 (24.5)	340.5 (25.0)		
Number of embryos transferred, n (%)			0.364	0.060			0.854	0.012
One embryo transferred	306 (29.5)	111 (32.3)			420.4 (30.4)	421.7 (30.9)		
Two embryo transferred	732 (70.5)	233 (67.7)			964.4 (69.6)	942.6 (69.1)		
Type of embryos transferred, n (%)			< 0.001	0.568			0.974	0.014
Cleavage-stage	305 (29.4)	111 (32.3)			419.4 (30.3)	421.7 (30.9)		
Blastocysts	696 (67.1)	164 (47.7)			859.3 (62.1)	837.6 (61.4)		
Cleavage-stage and blastocysts	37 (3.6)	69 (20.1)			106.1 (7.7)	104.9 (7.7)		

IPTW, inverse probability of treatment weighting;

HRT, hormone replacement therapy cycle;

GnRHa-HRT, hormone replacement therapy cycle with gonadotropin-releasing hormone agonist pre-treatment;

SD, standard deviation;

SMD, standardized mean difference.

After IPTW, there were no significant differences in terms of maternal age, body mass index, endometrial thickness, type of infertility and duration of infertility, number of transferred embryos, or embryo type between the two groups (all SMD<0.1 and P>0.05, **Table 1**).

### Pregnant outcomes

Pregnancy outcomes between the two groups were analyzed using univariate and multivariate logistic models, as shown in **Table 2**. The GnRHa-HRT group had a significantly higher live birth rate than the HRT group (OR 2.154, 95%CI 1.636~2.835, P<0.001), according to the results of both univariate and multivariate logistic regression analysis.

**Table 2:** Univariate and multivariate logistic analysis of pregnancy outcomes.

GnRHa-HRT group versus HRT group: Unadjusted and adjusted odds ratios (ORs)				
Outcome	Unadjusted OR (95% CI)	P-value	Adjusted OR (95% CI)	P-value
Live birth rate	2.111 (1.612~2.765)	< 0.001	2.154 (1.636~2.835)	< 0.001
Biochemical pregnancy	1.815 (1.376~2.395)	< 0.001	1.853 (1.398~2.457)	< 0.001
Clinical pregnancy	1.813 (1.382~2.378)	< 0.001	1.845 (1.401~2.430)	< 0.001
Miscarriage	0.445 (0.254~0.779)	0.004	0.432 (0.245~0.762)	0.004
Biochemical abortion	0.710 (0.385~1.312)	0.274	0.718 (0.390~1.325)	0.289
Ectopic pregnancy	1.226 (0.233~6.452)	0.809	1.171 (0.230~5.969)	0.849
Multiple pregnancy	1.803 (1.238~2.625)	0.002	1.908 (1.283~2.837)	0.001
Preterm delivery	0.926 (0.519~1.652)	0.794	0.977 (0.542~1.759)	0.937
Term delivery	1.759 (1.154~2.680)	0.009	1.745 (1.142~2.667)	0.010

HRT, hormone replacement therapy cycle;

GnRHa-HRT, hormone replacement therapy cycle with gonadotropin-releasing hormone agonist pre-treatment;

CI, confidence interval;

OR, odds ratio.

In multivariate logistic regression adjusted for confounding factors, the rate of miscarriage was significantly lower in the GnRHa-HRT group (OR 0.432, 95% CI 0.245~0.762, P=0.004). And the GnRHa-HRT group had a higher rate of biochemical pregnancy (OR 1.853, 95% CI 1.398~2.457, P<0.001), clinical pregnancy (OR 1.845, 95% CI 1.401~2.430; P<0.001), multiple pregnancy (OR 1.908, 95% CI 1.283~2.837, P=0.001), and term delivery (OR 1.745, 95% CI 1.142~2.667, P=0.010). The rate of biochemical abortion (OR 0.718, 95% CI 0.390~1.325, P=0.289), ectopic pregnancy (OR 1.171, 95% CI 0.230~5.969, P=0.849), and preterm delivery (OR 0.977, 95% CI 0.542~1.759, P=0.937) have no statistical difference between two groups.

## Discussion

This work showed that the GnRHa-HRT group had significantly improved pregnancy outcomes for male-factor infertility, including higher rates of live birth, biochemical pregnancy, and clinical pregnancy while reducing the miscarriage rate. Additionally, pregnancy complications such as biochemical pregnancy, ectopic pregnancy, and preterm birth did not significantly differ between the two groups.

The success of every ART mainly depends on the implantation of the embryo. Studies have shown that embryo quality, endometrial receptivity, and embryo-endometrial dialogue are three determining factors in successful implantation (34–38). We strictly performed the inclusion criteria in order to better rule out the influence of embryo quality. In addition, we excluded cases of female factors affecting infertility-related disorders. Therefore, we supposed that GnRH-a not only improves endometrial receptivity but also promotes dialogue between embryo and endometrium.

Endometrial thickness is one of the markers of endometrial receptivity. Before adjustment, The endometrium in the GnRHa-HRT group was thicker than that in the HRT group, which is in line with many previous researches (15, 39, 40). Some recent studies suggested that it is oestrogen supplementation without GnRH-a suppression that may lead to an increase in luteinizing hormone (LH), which ultimately has a detrimental effect on endometrial receptivity (23, 41, 42). Moreover, establishing a dialogue between endometrium and embryo, as well as immune tolerance/protection from the host, requires not only hormonal regulation but also several endogenous molecules produced by endometrium and/or embryo (34). A review found that male-factor infertility is related to spontaneous abortion rates (43). Several studies have shown that GnRH-a not only promotes the expression of protective endometrial receptivity markers such as LIF, MEIS1, and HOXA10 but also increases and develops pinopodes well (17) (44). In this study, the clinical pregnancy rate was higher and the miscarriage rate was lower in the GnRHa-HRT group, suggesting that GnRH-a plays an essential role in synchronizing endometrial and embryo development and promoting the dialogue between endometrium and embryo.

Although we excluded the low-quality embryo, male infertility can affect the quality of the embryo, which cannot be fully judged from the morphology of the embryo (45–48). Examples of factors that have negative effects on the quality of embryo include the effects of Y chromosome microdeletions, DNA fragmentation, sperm aneuploidy, the role of proseminins and histones, sperm epigenetic profiles, and sperm chromatin structure (45). We speculated that good endometrial-embryo dialogue can correct certain aspects of the embryo, making it easier to implant.

The following are the study’s limitations. 1) The impact of aneuploid embryos cannot be excluded. The evaluation of embryo quality in this study was mainly based on embryo morphology rather than preimplantation genetic testing for aneuploidy.2) Neonatal outcomes were not compared to fully assess the effect of GnRH-a on reproductive outcomes in male-factor infertility. 3) Although IPTW was used to balance baseline conditions, other unknown confounders, such as basal hormone levels, may also have affected the results. For more accurate findings, multicentre prospective clinical trials are required.

We have reason to assume that our findings are reliable given the IPTW-adjusted population baseline, multivariate logistic regression balancing confounders, and the substantial number of cases in this study. This paper will serve as a reference for the preparation of the endometrium for the treatment of female patients with male-factor infertility.

In conclusion, this study found that a regimen of GnRH-a preconditioning with HRT for endometrial preparation improved pregnancy outcomes in patients with male-factor infertility patients.

## Data availability statement

The raw data supporting the conclusions of this article will be made available by the authors, without undue reservation.

## Ethics statement

The studies involving human participants were reviewed and approved by the Ethics Committee of Shanghai General Hospital. Written informed consent for participation was not required for this study in accordance with the national legislation and the institutional requirements.

## Author contributions

JY and PC conducted the statistical analysis of the data and wrote the manuscript. YL was responsible for the collation of data. ML, LL, QX, JC, and LW were responsible for data acquisition and interpretation. ZZ contributed to the conception. MB contributed to the design of the work and rigorously revised the manuscript’s important intellectual content. All authors checked the lasted version. All authors contributed to the article and approved the submitted version.

## Funding

This work was supported by the National Natural Science Foundation of China (grants NO. 81902630, 81872111, and 81672562); the Shanghai Municipal Science and Technology Committee of Shanghai’s Excellent Academic Leader Program (grants NO. 19XD1423100), and the project of Outstanding Medical Doctor for ZZ.

## Acknowledgments

We would like to thank Jing Lu for her help in data analysis.

## Conflict of interest

The authors declare that the research was conducted in the absence of any commercial or financial relationships that could be construed as a potential conflict of interest.

## Publisher’s note

All claims expressed in this article are solely those of the authors and do not necessarily represent those of their affiliated organizations, or those of the publisher, the editors and the reviewers. Any product that may be evaluated in this article, or claim that may be made by its manufacturer, is not guaranteed or endorsed by the publisher.
